# Hydrogen (H_2_) Inhibits Isoproterenol-Induced Cardiac Hypertrophy via Antioxidative Pathways

**DOI:** 10.3389/fphar.2016.00392

**Published:** 2016-10-27

**Authors:** Yaxing Zhang, Jingting Xu, Zhiyuan Long, Chen Wang, Ling Wang, Peng Sun, Ping Li, Tinghuai Wang

**Affiliations:** ^1^Department of Physiology, Zhongshan School of Medicine, Sun Yat-sen UniversityGuangzhou, China; ^2^Department of Biomedical Engineering, Xinhua College, Sun Yat-sen UniversityGuangzhou, China

**Keywords:** hydrogen, β-adrenoceptor, cardiac hypertrophy, NADPH oxidase, reactive oxygen species, mitochondrial damage, MAPK

## Abstract

**Background and Purpose:** Hydrogen (H_2_) has been shown to have a strong antioxidant effect on preventing oxidative stress-related diseases. The goal of the present study is to determine the pharmacodynamics of H_2_ in a model of isoproterenol (ISO)-induced cardiac hypertrophy.

**Methods:** Mice (C57BL/6J; 8–10 weeks of age) were randomly assigned to four groups: Control group (*n* = 10), ISO group (*n* = 12), ISO plus H_2_ group (*n* = 12), and H_2_ group (*n* = 12). Mice received H_2_ (1 ml/100g/day, intraperitoneal injection) for 7 days before ISO (0.5 mg/100g/day, subcutaneous injection) infusion, and then received ISO with or without H_2_ for another 7 days. Then, cardiac function was evaluated by echocardiography. Cardiac hypertrophy was reflected by heart weight/body weight, gross morphology of hearts, and heart sections stained with hematoxylin and eosin, and relative atrial natriuretic peptide (ANP) and B-type natriuretic peptide (BNP) mRNA levels. Cardiac reactive oxygen species (ROS), 3-nitrotyrosine and p67 (phox) levels were analyzed by dihydroethidium staining, immunohistochemistry and Western blotting, respectively. For *in vitro* study, H9c2 cardiomyocytes were pretreated with H_2_-rich medium for 30 min, and then treated with ISO (10 μM) for the indicated time. The medium and ISO were re-changed every 24 h. Cardiomyocyte surface areas, relative ANP and BNP mRNA levels, the expression of 3-nitrotyrosine, and the dissipation of mitochondrial membrane potential (MMP) were examined. Moreover, the expression of extracellular signal-regulated kinase1/2 (ERK1/2), p-ERK1/2, p38, p-p38, c-Jun NH2-terminal kinase (JNK), and p-JNK were measured by Western blotting both *in vivo* and *in vitro*.

**Results:** Intraperitoneal injection of H_2_ prevented cardiac hypertrophy and improved cardiac function in ISO-infused mice. H_2_-rich medium blocked ISO-mediated cardiomyocytes hypertrophy *in vitro.* H_2_ blocked the excessive expression of NADPH oxidase and the accumulation of ROS, attenuated the decrease of MMP, and inhibited ROS-sensitive ERK1/2, p38, and JNK signaling pathways.

**Conclusion:** H_2_ inhibits ISO-induced cardiac/cardiomyocytes hypertrophy both *in vivo* and *in vitro*, and improves the impaired left ventricular function. H_2_ exerts its protective effects partially through blocking ROS-sensitive ERK1/2, p38, and JNK signaling pathways.

## Introduction

Heart failure is a global pandemic affecting an estimated 26 million people worldwide, posing an enormous burden to both individuals and society (Ambrosy et al., 2014). Heart failure is often preceded by left ventricular hypertrophy, which is characterized by an increase in the size of individual cardiac myocytes and re-expression of fetal cardiac genes, such as atrial natriuretic peptide (ANP) and B-type natriuretic peptide (BNP; Magga et al., 1998; Heineke and Molkentin, 2006). Although cardiac hypertrophy has traditionally been considered as an adaptive response required to sustain cardiac output in response to stresses, long-standing hypertrophy will eventually lead to congestive heart failure, arrhythmia, and sudden death (Frey and Olson, 2003).

Increasing evidence suggests that diverse pathophysiological stimuli, including neurohumoral activation [such as angiotensin II (ANG II) and β-adrenoceptor stimulation], hypertension, ischemic heart diseases, myocarditis, and diabetic cardiomyopathy, will contribute to cardiac hypertrophy and heart failure partially via inducing the production of excessive reactive oxygen species (ROS; Li et al., 2002; Zhang et al., 2007b; Zhang et al., 2015). The nicotinamide adenine dinucleotide phosphate (NADPH) oxidase and mitochondria have been proposed as primary sites of ROS generation (Dai et al., 2011a). ROS generated by NADPH oxidase was shown to stimulate and amplify mitochondrial ROS production and induce mitochondrial dysfunction, which can be reflected by the depression of mitochondrial membrane potential (MMP; Zorov et al., 2000; Dai et al., 2011a). The excessive accumulation of ROS subsequently activates downstream ROS-sensitive signaling pathways implicated in pathological cardiac hypertrophy. Therefore, blocking ROS will improve mitochondrial function and block downstream hypertrophic signaling, thus preventing the development of cardiac hypertrophy and progression to heart failure. Consistent with this notion, recent studies revealed that strategies targeted ROS and downstream signaling pathways modulated by ROS could be a better approach to improve cardiac hypertrophy (Burgoyne et al., 2012).

Molecule hydrogen (H_2_), which is a colorless, odorless, tasteless, and flammable gas, has attracted considerable attention for improving oxidative stress-related diseases (Ohta, 2015). We recently revealed that intraperitoneal injection of H_2_ protects against vascular hypertrophy induced by abdominal aortic coarctation (AAC) *in vivo*, and H_2_-rich medium attenuates proliferation and migration of vascular smooth muscle cells (VSMCs) stimulated by ANG II *in vitro* (Zhang et al., 2016). Moreover, H_2_ also has important role in protecting against heart diseases. Inhalation of H_2_ attenuates left ventricular remodeling induced by intermittent hypoxia (Hayashi et al., 2011; Kato et al., 2014), and improves cardiac hypertrophy after germinal matrix hemorrhage in neonatal rats (Lekic et al., 2011). However, the effects of H_2_ on cardiac hypertrophy induced by β-adrenoceptor stimulation and the related signaling mechanisms still remain unclear. The aims of this study are, therefore, to determine the effect of intraperitoneal injection of H_2_ on isoproterenol (ISO)-induced cardiac hypertrophy *in vivo*, and the effect of H_2_-rich medium on ISO-induced H9c2 cardiomyocytes hypertrophy *in vitro*, as well as to identify the molecular mechanisms that may be responsible for its putative effects.

## Materials and Methods

### Drugs and Chemicals

H_2_ (99.999%; Guang Zhou Guang Qi GAS Co., Ltd, Guangdong, China) was stored in the seamless steel gas cylinder, and it was injected into an aseptic soft plastic infusion bag (100 ml; CR Double-Crane Pharmaceuticals Co., Ltd, Anhui, China) under sterile conditions immediately before intraperitoneal injection. ISO (I5627, Sigma–Aldrich, St. Louis, MO, USA) was dissolved in normal saline (5 mg/10 ml) under sterile conditions immediately before subcutaneous injection, and dissolved in double distilled water as 10 mM stock solution 30 min before use. The antibodies against extracellular signal-regulated kinase 1/2 (ERK1/2), p-ERK1/2, p38, p-p38, c-Jun NH2-terminal kinase (JNK), and p-JNK, p67 (phox) were from Cell Signaling Technology (Danvers, MA, USA). The antibody against β-actin was from Santa Cruz Biotechnology (Santa Cruz, CA, USA). Anti-α-actin antibody was from Sigma–Aldrich (St. Louis, MO, USA). The antibody against 3-nitrotyrosine was from Abcam (Cambridge, MA, USA). JC-1 was from Beyotime Biotechnology (C2006, Jiangsu, China).

### Preparation of H_2_-rich Medium and Measurement of H_2_ Concentration

H_2_-rich medium was prepared as previously described (Zhang et al., 2016). The concentration of H_2_ was measured by MB-Pt reagent (generously provided by Ming Yan, Shanghai Nanobubble Technology Co., Ltd, Shanghai, China) as previously described (Zhang et al., 2016). The H_2_ concentration in our H_2_-rich medium was no less than 0.6 ppm (0.6–0.9 ppm).

### Cell Culture and Treatment

H9c2 rat cardiac myoblasts (a cardiomyoblast cell line derived from embryonic rat heart tissue; generously provided by Prof. Hongliang Li, Wuhan University, China) were grown in DMEM containing 5.5 mM glucose as described previously (Jeong et al., 2009). To induce hypertrophy, cells were serum starved for 18 h in DMEM containing 1% FBS, and then treated with 10 μM ISO for 48 h (Jeong et al., 2009). In order to investigate the effect of H_2_ on the blockage of ISO-induced hypertrophy, H_2_-rich medium was added 30 min before ISO administration, the medium, and ISO were re-changed every 24 h, and cardiomyocytes hypertrophic response was examined after 48 h of ISO challenge (Jeong et al., 2009).

### Animal Model of Cardiac Hypertrophy and Treatment Protocol

The C57BL/6J mice (aged 8–10 weeks, male) were obtained from the Laboratory Animal Center of Sun Yat-sen University. The animals were housed with 12-h light–dark cycles and allowed to obtain food and water *ad libitum*. All experimental procedures and protocols were approved by Institutional Animal Care and Use Committee (Zhongshan School of Medicine, Sun Yat-sen University), and conformed to the *Guide for the Care and Use of Laboratory Animals* published by the National Institutes of Health (NIH publication NO. 85-23, revised 1996).

Cardiac hypertrophy was induced by subcutaneous injection of ISO (0.5 mg/100g/day) for 7 days as previously revealed (Tshori et al., 2006). Mice were randomly assigned to four groups: Control (Con) group (*n* = 10), ISO group (*n* = 12), ISO plus H_2_ group (*n* = 12), and H_2_ group (*n* = 12). H_2_ was given at the dose of 1 ml/100g/day by intraperitoneal injection as previously described (Huang et al., 2013; Zhang et al., 2016). Mice in ISO plus H_2_ group and H_2_ group received H_2_ consecutively for 7 days before receiving ISO, and continued for another 7 days. On the 8th day, mice in ISO group, and ISO plus H_2_ group received ISO for 7 days until animals were sacrificed on the 15th day. After sacrifice, hearts were excised, rinsed with ice-PBS, and blotted dry. Hearts were weighed; the heart weight/body weight (HW/BW) ratios were calculated and expressed as milligrams HW per gram BW. Then hearts were snap frozen in liquid nitrogen within minutes and stored at -80°C until analyzed.

### Echocardiography

Transthoracic echocardiography was performed to assess left ventricular function before sacrificed on the 15th day in a blinded manner. Mice were anesthetized with 1.5–2% isoflurane, and hearts were visualized using a RMV707B (30 M Hz) scan-head interfaced with a Vevo-2100 high frequency ultrasound system (VisualSonics Inc., Toronto, Canada) at least three times for each animal indicated (Webb et al., 2010).

### Histological Analysis

Hearts were excised, washed with ice-PBS, fixed in 10% buffered formalin, and cut transversely close to the apex cordis to visualize the left and right ventricles. Several sections of heart (4–5 μm thickness) were prepared and stained with hematoxylin and eosin (H&E) for histopathology and then visualized by light microscopy.

### Immunohistochemistry

For immunostaining, anti-sarcomeric α-actin antibody was used to assess the cell surface area of H9c2 cardiomyocytes as described previously (Akimoto et al., 1996). To assess 3-nitrotyrosine levels in heart, which can reflect formation of ONOO–, primary antibody against 3-nitrotyrosine (1:50) was used as previously described (Zhang et al., 2011).

### Measurement of MMP

Mitochondrial membrane potential was determined by the dye 5,5′,6,6′-tetrachloro-1,1′,3,3′-tetraethylbenzimidazolcarbo-cyanine iodide (JC-1) as previously described with slight modification (Cossarizza et al., 1993). Briefly, the treated cells were washed with PBS, and then incubated with JC-1 staining dye (culture medium: JC-1 working dye = 1:1) at 37°C in the dark for 20 min and rinsed three times with cold PBS, and analyzed by fluorescence microscope (Axio Observer Z1, Carl Zeiss. Inc.). The JC-1 aggregates, which was accumulated in the inner membrane of mitochondria, emitted red fluorescence and represented the high MMP, while green fluorescence reflected JC-1 monomer which entered in the cytosol following mitochondrial membrane depolarization. When mitochondria is damaged, the red/green ratio decreases. The ratio of JC-1 aggregates to monomer (red/green) intensity for each region was calculated by Image-Pro Plus software (version 6.0).

### qRT-PCR

Total mRNA was extracted from left ventricles and H9c2 cardiomyocytes using TRIZol reagent (15596-026, Invitrogen) according to the manufacturer’s instruction, and cDNA was synthesized using oligo (dT) primers with the Transcriptor First Strand cDNA Synthesis Kit (PrimeScript^TM^ RT Master Mix, Takara). Selected gene differences were confirmed by qRT-PCR using SYBR green (SYBR^®^ Premix Ex Taq^TM^, Takara). The target gene expression was normalized to GAPDH gene expression. The primers for qRT-PCR are shown in **Table 1**.

**Table 1:** The primers for qRT-PCR.

Name	Forward primer sequence (5′–3′)	Reverse primer sequence (5′–3′)
M-GAPDH	GGTTGTCTCCTGCGACTTCA	TGGTCCAGGGTTTCTTACTCC
R-GAPDH	GACATGCCGCCTGGAGAAAC	AGCCCAGGATGCCCTTTAGT
M-ANP	GTCTTGCCTCTCCCACTCTG	TTCGTCCTTGGTGCTGAAGT
R-ANP	GGGAAGTCAACCCGTCTCA	GGCTCCAATCCTGTCAATCC
M-BNP	TCTGGGACCACCTTTGAAGT	ATGTTGTGGCAAGTTTGTGC
R-BNP	CTCCAGAACAATCCACGATG	ACAGCCCAAGCGACTGACT

### Western Blotting

Western blotting was performed as previously described (Zhang et al., 2016). The membranes were incubated with primary (1:2000) and secondary (1:2000) antibodies by standard techniques. Immunodetection was accomplished using enhanced chemiluminescence (ChemiDoc XRS+ System, Bio-Rad, Hercules, CA, USA).

### Assessment of Cardiac ROS Levels

Cardiac total ROS was stained with dihydroethidium (DHE, D-23107; Invitrogen) on fresh frozen sections as previously described (Zhang et al., 2014). Images were immediately acquired using confocal microscopy (Leica Model SPE, Leica Imaging Systems Ltd) using λ_ex_ 405 nm laser excitation.

### Statistical Analysis

Data are expressed as mean ± SD. Differences among groups were tested by one-way ANOVA. Comparisons between two groups were performed by unpaired Student’s *t*-test. A value of *P* < 0.05 was considered to be significantly different.

## Results

### H_2_ inhibited Cardiac Hypertrophy *In vivo*

**FIGURE 1:**
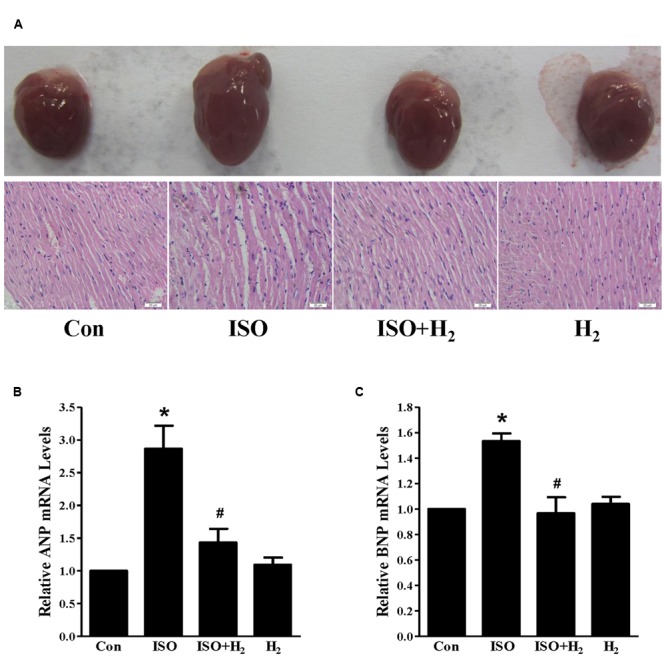
**Effects of hydrogen (H_2_) on cardiac hypertrophy induced by isoproterenol (ISO) *in vivo*. (A)** Gross morphology of hearts (top) and heart sections stained with H&E (bottom) after 1 week of ISO infusion with or without H_2_ at the dose of 1 ml/100g/day. **(B)** The relative mRNA expression of hypertrophic marker atrial natriuretic peptide (ANP) to GAPDH (*n* = 3). **(C)** The relative mRNA expression of hypertrophic marker B-type natriuretic peptide (BNP) to GAPDH (*n* = 3). *^∗^P* < 0.05 vs. Control (Con) and *^#^P* < 0.05 vs. ISO. Scale bar: 20 μm.

In order to investigate the effects of H_2_ on cardiac hypertrophy, ISO was used to induce cardiac hypertrophy in mice. As expected, mice with chronic ISO infusion exhibited cardiac hypertrophy compared to the control group, as indicated by the gross morphology of hearts, heart sections stained with H&E (**Figure 1A**). The hypertrophic marker gene ANP and BNP mRNA levels (**Figures 1B,C**, *P* < 0.05 vs. Con), and HW/BW ratio (**Table 2**, *P* < 0.05 vs. Con) were also increased. Pretreatment with H_2_ (intraperitoneal injection) at the dose of 1 ml/100g/day reversed these hypertrophic responses (**Figure 1**; **Table 2**, *P* < 0.05 vs. ISO). Moreover, H_2_ injection alleviated the impaired left ventricular function, as evidenced by decreasing left ventricular end-systolic diameter (LVESD), left ventricular end-diastolic diameter (LVEDD), and increasing fractional shortening (FS%; **Table 2**, *P* < 0.05 vs. ISO). However, there were no significant changes between control group and H_2_ group. Collectively, these data suggested that H_2_ injection prevented the development of ISO-induced cardiac hypertrophy and preserved cardiac function *in vivo*.

**Table 2:** Effects of hydrogen on cardiac dysfunction induced by isoproterenol (ISO) *in vivo.*

Parameter	Con	ISO	ISO+H_2_	H_2_
Number (*n*)	10	12	12	12
HW/BW (mg/g)	4.72 ± 0.08	5.81 ± 0.07^∗^	5.09 ± 0.14*^#^*	4.69 ± 0.06
LVEDD (mm)	3.24 ± 0.10	3.71 ± 0.06^∗^	3.45 ± 0.01*^#^*	3.26 ± 0.08
LVESD (mm)	2.05 ± 0.05	2.52 ± 0.07^∗^	2.35 ± 0.04*^#^*	2.12 ± 0.07
FS (%)	35.99 ± 0.13	31.56 ± 0.19^∗^	33.30 ± 0.45*^#^*	35.68 ± 0.26

*Hydrogen was given at the dose of 1 ml/100g/day by intraperitoneal injection. Heart weight, HW; Body weight, BW; LVEDD, left ventricular end-diastolic diameter; LVESD, left ventricular end-systolic diameter. *^∗^P* < 0.05 vs. Con and *^#^P* < 0.05 vs. ISO.*

### H_2_ attenuated Cardiomyocyte Hypertrophy *In vitro*

**FIGURE 2:**
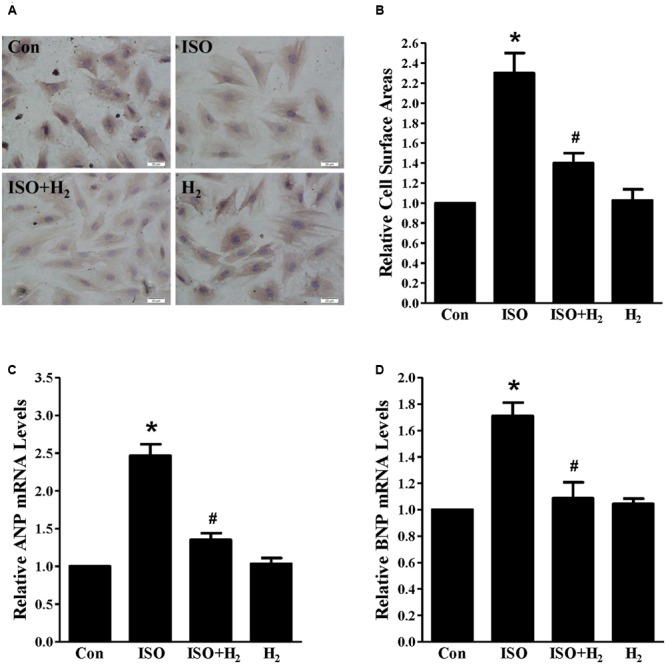
**Effects of H_2_-rich medium on cardiomyocytes hypertrophy induced by ISO *in vitro*. (A)** Photomicrographs of morphological change induced by ISO with or without H_2_-rich medium. **(B)** Bar graph shows the relative cell surface area of cardiomyocytes stimulated by ISO with or without H_2_-rich medium. **(C)** The relative mRNA levels of hypertrophic marker ANP to GAPDH (*n* = 4). **(D)** The relative mRNA levels of hypertrophic marker BNP to GAPDH (*n* = 4). *^∗^P* < 0.05 vs. Con and *^#^P*< 0.05 vs. ISO. Scale bar: 20 μm.

As the heart primarily consists of cardiomyocyte and fibroblast, therefore, we investigated whether H_2_ could target cardiomyocyte for hypertrophic inhibition. H_2_-rich medium and H9c2 cardiomyocytes were used for *in vitro* studies. First, we used CCK8 to investigate the possible cytotoxity of H_2_-rich medium on H9c2 cardiomyocyte. H_2_ was shown to be non-cytotoxic for cardiomyocyte treating with H_2_-rich medium for 48 h (data not shown). After 48 h of ISO stimulation, cardiomyocyte surface areas, and the hypertrophic marker gene ANP and BNP mRNA levels were significantly increased in H9c2 cardiomyocyte (**Figures 2A–D**, *P* < 0.05 vs. Con). H_2_-rich medium attenuated these hypertrophic responses of H9c2 cardiomyocyte (**Figures 2A–D**, *P* < 0.05 vs. ISO). These data indicated that H_2_ could also inhibit ISO-induced cardiomyocyte hypertrophy *in vitro*.

### H_2_ Blocked the Excess ROS Accumulation and Mitochondrial Damage

**FIGURE 3:**
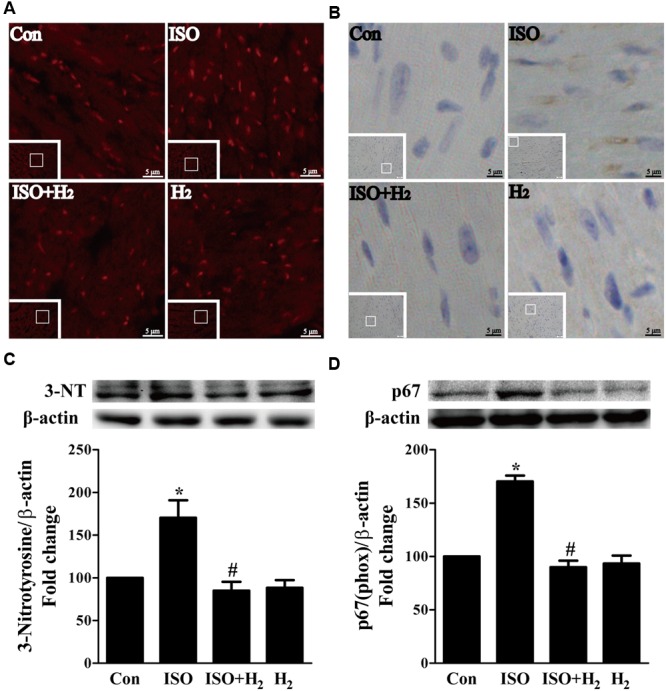
**Effects of H_2_ on the generation of Reactive oxygen species (ROS) induced by ISO both *in vivo* and *in vitro*. (A)** Total ROS was stained with dihydroethidium (DHE) in the heart infused by ISO 1 week with or without H_2_ at the dose of 1 ml/100g/day. **(B)** Cardiac 3-nitrotyrosine (3-NT) was stained by immunohistochemistry in different groups. **(C)** Representative Western blotting and quantification of 3-NT to β-actin in H9c2 cardiomyocytes stimulated by ISO for 5 min with or without H_2_-rich medium for 30 min pretreatment (*n* = 4). **(D)** Representative Western blotting and quantification of p67 (phox) to β-actin in the hearts (*n* = 4). *^∗^P* < 0.05 vs. Con and *^#^P* < 0.05 vs. ISO. Scale bar: 5 μm.

ROS play a critical role in the development of cardiac hypertrophy and heart failure (Burgoyne et al., 2012). ROS levels were increased in the left ventricular of ISO-infused mice compared with control mice, and this increase was inhibited by pretreatment with H_2_ at the dose of 1ml/100g/day (**Figure 3A**). Moreover, another oxidative stress marker, 3-nitrotyrosine (3-NT), which reflects the formation of ONOO–, was also upregulated by ISO stimuli, and suppressed by H_2_ (**Figure 3B**). To confirm these *in vivo* findings, we evaluated the effects of H_2_-rich medium on the levels of 3-NT stimulated by ISO *in vitro*. The accumulation of 3-NT was increased after ISO stimulation, while H_2_-rich medium attenuated this effects (**Figure 3C**, *P* < 0.05).

**FIGURE 4:**
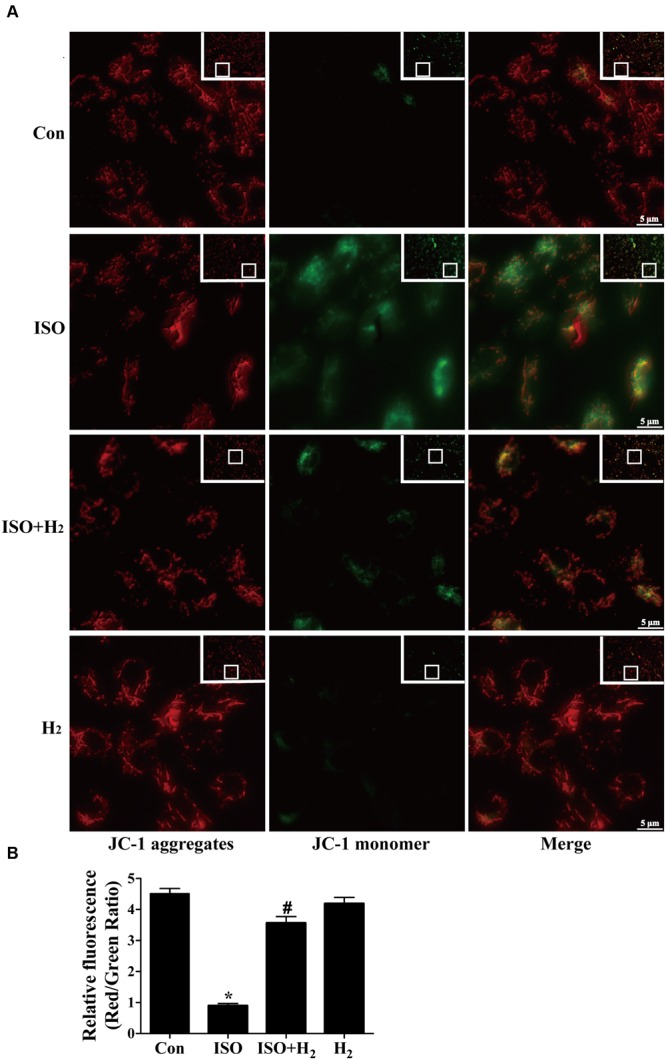
**Effects of H_2_-rich medium on ISO-induced depression of MMP *in vitro*.** After stimulated by ISO for 24 h with or without H_2_-rich medium for 30 min pretreatment, MMP was measured by JC-1 staining followed by photofluorography **(A)**. The quantification of the fluorescence intensity (red/green ratio) for each treatment was calculated by Image-Pro Plus software **(B)** (*n* = 4). ^∗^*P* < 0.05 vs. Con and *^#^P* < 0.05 vs. ISO. Scale bar: 5 μm.

To further understand the mechanism of H_2_ in blocking ROS accumulation, we tested the NADPH oxidase subunit p67 (phox) expression. Immunoblotting revealed the expression of p67 (phox) was increased in left ventricular of ISO-infused mice, and this increase was alleviated by H_2_ (**Figure 3D**, *P* < 0.05). As we have mentioned above, NADPH oxidase-derived ROS can stimulate and amplify mitochondrial ROS production and induce mitochondrial dysfunction (Zorov et al., 2000; Dai et al., 2011a), and these can be reflected by the change of MMP. ISO induced the depression of MMP, as indicated by high levels of green fluorescence and low levels of red fluorescence. Interestingly, H_2_-rich medium blocked the depression of MMP induced by ISO (**Figures 4A,B**, *P* < 0.05). Therefore, these data indicated that H_2_ inhibited the excess ROS accumulation following ISO stimuli through attenuating NADPH oxidase expression and mitochondrial damage.

### H_2_ suppressed Mitogen-Activated Protein Kinases (MAPKs) Signaling *In vivo* and *In vitro*

**FIGURE 5:**
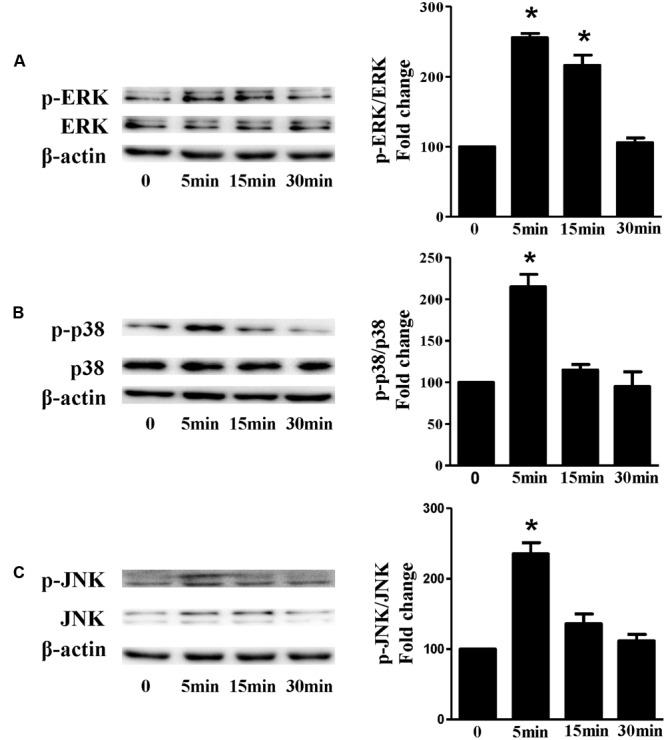
**The time-dependent effects of ISO on MAPKs activation *in vitro*.** Representative Western blot and quantification of ERK1/2 phosphorylation **(A)**, or p38 phosphorylation **(B)**, or JNK phosphorylation **(C)** to their total protein expressions, respectively, *n* = 4. *^∗^P* < 0.05 vs. 0 min.

**FIGURE 6:**
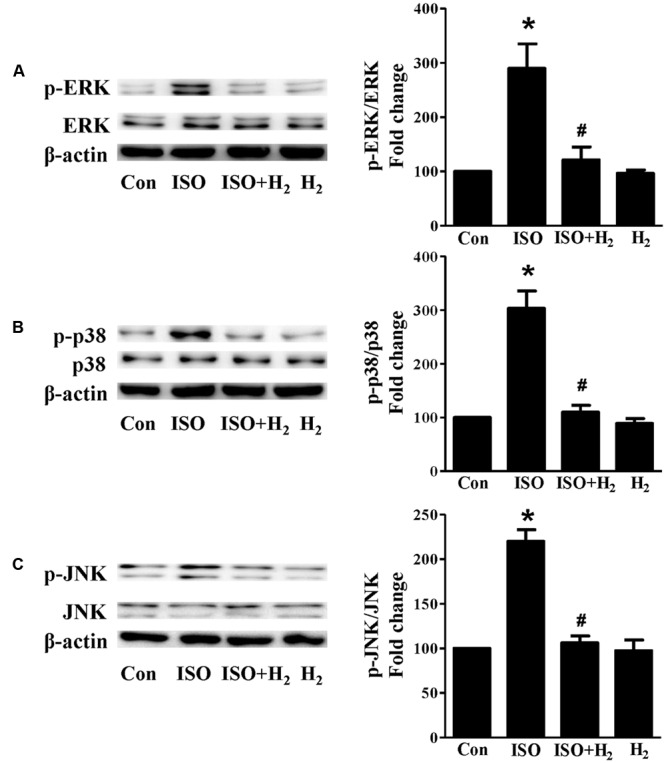
**Effects of H_2_-rich medium on ISO-mediated MAPKs signaling activation *in vitro*.** Representative Western blot and quantification of ERK1/2 phosphorylation **(A)**, p38 phosphorylation **(B)**, and JNK phosphorylation (**C**) to their total protein expressions, respectively, *n* = 4. *^∗^P* < 0.05 vs. Con and *^#^P* < 0.05 vs. ISO.

**FIGURE 7:**
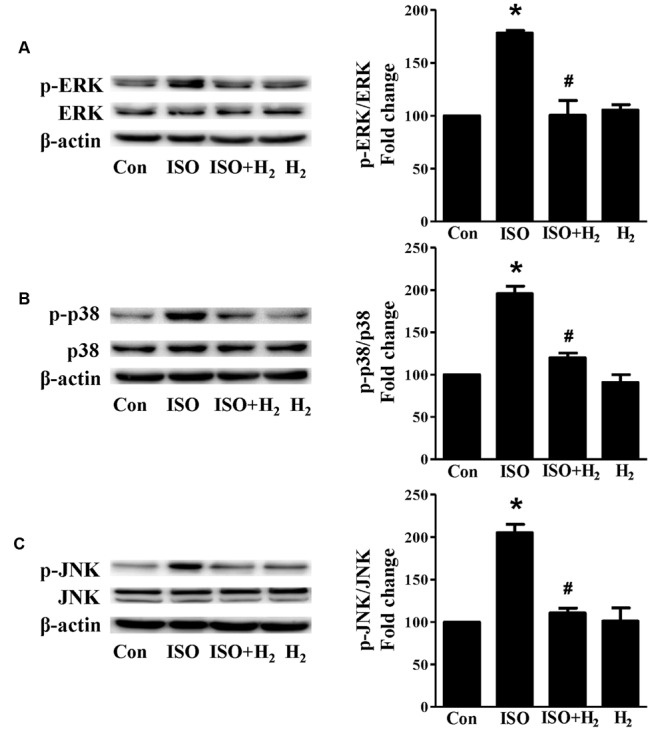
**Effects of H_2_ (1 ml/100g/day) on MAPKs signaling activation induced by ISO *in vivo*.** Representative Western blotting and quantification of ERK1/2 phosphorylation **(A)**, or p38 phosphorylation **(B)**, or JNK phosphorylation **(C)** to their total protein expressions, respectively, *n* = 4. *^∗^P* < 0.05 vs. Con and *^#^P* < 0.05 vs. ISO.

Based on the inhibitory effect of H_2_ on the ISO-induced excess accumulation of ROS *in vitro* and *in vivo*, we further investigated its effect on the downstream hypertrophic targets, such as mitogen-activated protein kinases (MAPKs) signaling pathways. Following ISO stimuli, the phosphorylation of ERK1/2, p38 MAPK (p38), and c-Jun NH2-terminal kinase (JNK) were increased to the high level at 5 min, and came to the base line at 30 min (**Figure 5**, *P* < 0.05 vs. 0 min). These enhanced activation of MAPKs could be blocked by H_2_-rich medium *in vitro* (**Figure 6**; *P* < 0.05 vs. ISO). Similarly, the activation of MAPKs were enhanced in the hearts of ISO-infused mice compared with control group (**Figure 7**, *P* < 0.05 vs. Con). Such changes were inhibited by pretreatment with H_2_ *in vivo* (**Figure 7**, *P* < 0.05 vs. ISO). Thus, H_2_ suppressed the enhanced phosphorylation of ERK1/2, p38, and JNK to alleviate ISO-mediated cardiac hypertrophy *in vivo* and cardiomyocyte hypertrophy *in vitro.*

## Discussion

The present study demonstrates that intraperitoneal injection of H_2_ protects against ISO-induced cardiac hypertrophy and dysfunction *in vivo* and H_2_-rich medium attenuates ISO-mediated cardiomyocyte hypertrophy *in vitro.* The cardioprotection of H_2_ is mediated by direct interruption of NADPH oxidase expression and alleviating mitochondrial damage, these lead to inhibit the accumulation of ROS, and subsequently block downstream ERK1/2, p38, and JNK signaling.

H_2_ has been emerged as an important blocker of heart diseases by various given manners. H_2_ inhalation attenuates intermittent hypoxia (Hayashi et al., 2011; Kato et al., 2014), or ischemia/reperfusion (Hayashida et al., 2008), or germinal matrix hemorrhage-induced left ventricular remodeling (Lekic et al., 2011). Drinking H_2_-rich water blocks cardiac fibrosis induced by left kidney artery ischemia/reperfusion injury (Zhu et al., 2011). H_2_-rich saline injection also inhibits ischemia/reperfusion (Sun et al., 2009), or hypertension-mediated cardiac remodeling (Wang et al., 2011; Yu and Zheng, 2012). However, the effects of intraperitoneal injection of H_2_ on cardiac hypertrophy induced by β-adrenoceptor stimulation have not yet been clarified. In this study, we prepared H_2_-rich medium, and developed new methods for giving H_2_ *in vivo* by intraperitoneal injection of H_2_, and we find that H_2_ not only attenuates ISO-induced cardiomyocyte hypertrophy *in vitro* and cardiac hypertrophy *in vivo*, but also improves the impaired cardiac function. As we have mentioned above, diabetic cardiomyopathy is also a contributor to cardiac hypertrophy and heart failure. H_2_-rich saline has been reported to improve early neurovascular dysfunction (Feng et al., 2013) and erectile dysfunction (Fan et al., 2013) in a streptozotocin-induced diabetic rat model. However, the effect of H_2_ on diabetic cardiomyopathy is still under investigation. It has been reported that the gasotransmitter hydrogen sulfide (H_2_S) protects against pressure overload-mediated (Kondo et al., 2013) or arteriovenous fistula (AVF)-induced heart failure (Mishra et al., 2010). A question raised here is that whether the reciprocal interaction between H_2_ and H_2_S exists during their regulation of cardiac hypertrophy.

The excess activation of ROS has been shown to contribute to the development of cardiac hypertrophy (Li et al., 2002; Zhang et al., 2005, 2007b; Burgoyne et al., 2012). In this study, we reveal that H_2_ blocks ROS accumulation induced by β-adrenoceptor stimulation both *in vitro* and *in vivo.* The inhibitory effects of H_2_ on ROS also have been reported in various animal models, such as heart ischemia/reperfusion injury (Zhang et al., 2011; Noda et al., 2013; Shinbo et al., 2013), brain injury (Ohsawa et al., 2007; Liu et al., 2011; Wang et al., 2012), renal injury (Li et al., 2016), chemotherapy-induced ovarian injury (Meng et al., 2015), metabolic syndrome (Song et al., 2013), etc. NADPH oxidase and mitochondria have been proposed as primary sites of ROS generation (Dai et al., 2011a). ROS produced by NADPH oxidase has the ability to stimulate and amplify mitochondrial ROS generation and induce mitochondrial dysfunction (Zorov et al., 2000; Dai et al., 2011a). Therefore, tyrosine kinase FYN interacts with the C-terminal domain of NOX4, and phosphorylates the tyrosine 566 on NOX4, thereby inhibiting apoptosis in the heart and preventing cardiac remodeling after pressure overload (Matsushima et al., 2016). Overexpression of catalase targeted to mitochondria, but not the overexpression of wild-type peroxisomal catalase, protects against ANG II-induced cardiac hypertrophy, fibrosis and mitochondrial damage, as well as heart failure induced by overexpression of Gαq (Dai et al., 2011b). We found that H_2_ inhibits ISO-induced NADPH oxidase subunit p67 expression, and suppresses the dissipation of MMP.

The excessive accumulation of ROS subsequently transmits signals to downstream ROS-sensitive signaling pathways, such as ERK1/2 (Li et al., 2002; Dai et al., 2011b), p38 MAPK (Li et al., 2002; Dai et al., 2011a), and JNK (Li et al., 2002; Kimura et al., 2005; Zhang et al., 2007a), NF-κB (Hirotani et al., 2002), PI3K/Akt (Sundaresan et al., 2009; Wang et al., 2013), and autophagy related signaling (Dai et al., 2011b), to induce pathological cardiac hypertrophy. Our results indicate that H_2_ markedly blocks ISO-induced ERK1/2, p38 and JNK activation *in vivo* and *in vitro*. These findings confirm that the anti-hypertrophic effect of H_2_ is partially achieved through blocking ROS-dependent MAPKs signaling. Yu Yongsheng et al. has reported that H_2_-rich saline inhibits cardiac hypertrophy in spontaneous hypertensive rats (SHRs) *via* blocking NF-κB activity (Yu and Zheng, 2012). H_2_-rich saline reduces myocardial reperfusion injury and improves heart function through down-regulating the expression of Akt and GSK3β (Yue et al., 2015), and blocking autophagy in myocardial tissue (Pan et al., 2015). However, whether PI3K/Akt, and autophagy signaling are related to the protective effects of H_2_ injection on pathological cardiac hypertrophy still needs further investigation.

## Conclusion

Our study demonstrated that intraperitoneal injection of H_2_ attenuated β-adrenoceptor agonist (ISO)-mediated cardiac hypertrophy and dysfunction *in vivo*, and H_2_-rich medium blocked ISO-induced cardiomyocyte hypertrophic responses *in vitro*. Our results suggested that H_2_ exerted anti-hypertrophic activity, at least in part, *via* alleviating NADPH oxidase expression and inhibiting the depression of MMP, and thus blocked ROS-sensitive MAPK signaling pathways.

## Author Contributions

Conceived and designed the experiments: YZ and TW. Performed the experiments: YZ, JX, ZL, and CW. Analyzed the data: YZ and JX. Contributed reagents/materials/analysis tools: LW, PS, and PL.

## Conflict of Interest Statement

The authors declare that the research was conducted in the absence of any commercial or financial relationships that could be construed as a potential conflict of interest.
